# An audit of knowledge, attitude and sexual practise of HIV positive patients in Beaumont Hospital.

**DOI:** 10.1186/1753-6561-9-S7-A8

**Published:** 2015-10-27

**Authors:** David McMahon, Samuel McConkey

**Affiliations:** 1 Royal College of Surgeons in Ireland, Dublin 2, Ireland; 2 Beaumont Hospital, Dublin 9, Ireland

## Background

This study explores the effectiveness of information provision in the HIV clinic in Beaumont Hospital, determining whether the patients understand the information provided and change their beliefs and practices as a result.

## Methods

Patients were asked a series of questions in a structured interview. The information was then analysed on a population basis by ethnicity and viral load.

## Results

684 questions were asked to ascertain the patient's knowledge of HIV transmission with an 82% (n=559) rate of correct answers. 72% (n=37) of those who had sex in the past year in this study stated that they had not had sex without a condom in that period.

## Conclusions

There is room for improvement in knowledge of transmission in the clinic. An 8% gap existed between Irish patients (89%) and all other patients (81%) in the area of HIV transmission. The understanding that antiretroviral drugs can aid in preventing HIV transmission is lacking in many patients. Many patients have changed their sexual practices while attending the clinic to protect both themselves and their partners.

